# Leveraging Plasma-Activated Seawater for the Control of Human Norovirus and Bacterial Pathogens in Shellfish Depuration

**DOI:** 10.3390/foods13060850

**Published:** 2024-03-11

**Authors:** Annamaria Pandiscia, Patrizio Lorusso, Alessio Manfredi, Gloria Sánchez, Valentina Terio, Walter Randazzo

**Affiliations:** 1 Department of Preservation and Food Safety Technologies, Institute of Agrochemistry and Food Technology (IATA-CSIC), Av. Agustín Escardino 7, Paterna, 46980 Valencia, Spain; 2 Department of Veterinary Medicine, University of Bari, Provincial Road to Casamassima Km 3, 70010 Valenzano, Italy

**Keywords:** norovirus, vibrio, bivalve mollusks, inactivation, food safety, bacteriophages, food processing technology, water quality, contamination

## Abstract

Cold plasma is a promising alternative for water treatment owing to pathogen control and a plethora of issues in the agriculture and food sectors. Shellfish pose a serious risk to public health and are linked to large viral and bacterial outbreaks. Hence, current European regulations mandate a depuration step for shellfish on the basis of their geographical growth area. This study investigated the inactivation of relevant viral and bacterial pathogens of three plasma-activated seawaters (PASWs), and their reactive oxygen and nitrogen species (RONS) composition, as being primarily responsible for microbial inactivation. Specifically, F-specific (MS2) and somatic (φ174) bacteriophage, cultivable surrogate (murine norovirus, MNV, and Tulane virus, TV), and human norovirus (HuNoV GII.4) inactivation was determined using plaque counts and infectivity assays, including the novel human intestinal enteroid (HIE) model for HuNoV. Moreover, the kinetic decay of *Escherichia coli, Salmonella* spp., and *Vibrio parahaemolyticus* was characterized. The results showed the complete inactivation of phages (6–8 log), surrogates (5–6 log), HuNoV (6 log), and bacterial (6–7 log) pathogens within 24 h while preventing cytotoxicity effects and preserving mussel viability. Nitrites (NO_2_^−^) were found to be mostly correlated with microbial decay. This research shows that PASWs are a suitable option to depurate bivalve mollusks and control the biohazard risk linked to their microbiological contamination, either viral or bacterial.

## 1. Introduction

The global demand for innovative technologies in the food sector is rising, driven by regulatory reviews focusing on sustainability, long-term eco-safety, and human safety, prompting food business operators (FBOs) to seek new technology interventions. Over the last few decades, plasma science and technology has been increasingly investigated to solve the challenges facing by the agriculture and food industry [1,2,3,4]. Non-thermal atmospheric plasma, a technology with proven antibacterial effects [5,6,7,8,9], produces plasma-activated water (PAW) upon application to water as a result of the chemical reaction between the ionized gas and the adjacent liquid [10]. The primary factor responsible for such microbial inactivation is a highly oxidizing environment with reduced pH and reactive oxygen and nitrogen species (RONS), such as hydrogen peroxide (H_2_O_2_), nitrate (NO_3_^−^), and nitrite ions (NO_2_^−^) [4,10,11,12,13,14,15,16,17]. Due to the absence of chemical residues (e.g., trihalomethanes derived from chlorine disinfection) and the absence of a negative environmental impact, along with diversity in its modes of application, PAW has been observed as a sustainable candidate for use as a sanitizer on food surfaces, food preparation surfaces, and processing equipment [18,19,20,21,22,23].

Low-temperature treatments, the use of air as the working gas, short treatment times, and remarkable antimicrobial efficacy represent the most promising features of applying PAW technology in the food industry. However, challenges such as elevated investment costs, limited knowledge regarding effective doses for various combinations of food matrices and pathogens, and the understudied impact on food quality all present opportunities for future research [1,24].

Based on their high filter-feeding activities, shellfish can bioaccumulate several foodborne pathogens, representing a serious risk to public health since they are often consumed raw or lightly cooked [25]. Epidemiological investigations, microbiological analyses, and scientific evidence frequently confirm the presence of different microbial pathogens in bivalve mollusks, with human norovirus (HuNoV) and *Vibrio* spp. being the etiological agents most often implicated in outbreaks [12]. In Europe, the Rapid Alert System for Food and Feed (RASFF) portal recorded seventy-five notifications of bivalve mollusks contaminated by pathogenic microorganisms in 2023. Among them, HuNoV was linked to more than thirty-five alerts, while *Salmonella* spp. and *Escherichia coli* were reported in ten alerts and *Vibrio* spp. were reported in four [26]. In 2019, a European baseline survey found that 34.5% and 10.8% of oysters from production areas and dispatch centers were contaminated with HuNoV, respectively [27].

HuNoV causes acute diarrheal gastroenteritis frequently linked to large outbreak sizes with a high number of human cases.

*Vibrio* spp. cause infections in humans via the oral route through the ingestion of contaminated water or raw or undercooked contaminated seafood (in particular, *Vibrio parahaemolyticus* and *V. cholerae*) [28,29]. *V. parahaemolyticus* is the most common pathogen causing seafood-borne illnesses in many countries, and the strains harboring the *tdh* (encoding thermostable direct hemolysin) and *trh* (encoding tdh-related hemolysin) genes are pathogenic in humans [30]. *Vibrio* spp. are one of the zoonotic agents listed in Annex I of Directive 2003/99/EC to be monitored according to the epidemiological situation [31,32]. Moreover, FBOs must control the levels of fecal bacterial indicators such as *E. coli* and *Salmonella* spp. [33]. Salmonellosis is the second most reported zoonosis in humans followed by *Listeria monocytogenes* and Shiga toxin-producing *E. coli* (STEC), including the O157, O26, O103, O146, O145, and O91 serogroups [34].

Considering this risk, the European Union (EU) laid down the criteria for the classification of shellfish harvesting areas and determined the level of post-harvest treatment required before shellfish can be considered suitable for human consumption. The harvesting areas are classified into three sanitary levels (A, B, and C) (EU/2019/627), and current European regulations mandate a depuration process for mollusks harvested in B and C areas, ensuring compliance with the microbiological criteria included in EC Reg. 1441/2007 [33,35]. Specifically, while shellfish from A areas do not undergo depuration (*E. coli* colony-forming units (CFUs) ≤ 230/100 g shellfish in 80% of samples), increased levels of contamination are expected in B (*E. coli* CFUs ≤ 4600/100 g shellfish in 90% of samples) and C areas (*E. coli* CFUs ≤ 46,000/100 g shellfish in 90% of samples), and a depuration step is enforced [36]. Nevertheless, outbreaks still occur, and the scientific community agrees on the inadequacy of current commercial shellfish depuration processes for HuNoVs and *Vibrio* spp. [25,37,38].

To add complexity to this scenario, the standard procedure to detect HuNoV in bivalve mollusks (ISO 15216-1:2017) [39] relies on molecular methods, which do not provide information on viral infectivity. Indeed, the lack of a reliable propagation model for HuNoV has been a significant roadblock to studying its stability, persistence, and inactivation [40]. To overcome this limitation, bacteriophages (e.g., MS2, and φ174) and cultivable surrogates (e.g., murine norovirus, MNV, and Tulane virus, TV) have been historically used, even though they provide biased data and still represent an indirect estimation for the actual human viral pathogen [41,42,43]. Specifically, F-specific (e.g., MS2) and somatic (e.g., φ174) bacteriophages have been commonly used as viral surrogates for viral inactivation and as indicators for fecal contamination in Europe and the USA [44,45]. Recently, a novel three-dimensional cell culture technique based on stem cell-derived human intestinal enteroids (HIEs) has been described and confirmed to support the replication of HuNoVs [46,47], enabling the first inactivation studies [48,49,50,51].

While PAW has been applied in various foods, there are a lack of studies exploring the production of plasma-activated seawater and its application for seafood depuration [52]. Given that electrical conductivity is directly associated with the number of ions generated in solution following plasma treatment, the salinity of seawater presents additional challenges for the production of RONS and maintaining their stability over time.

Taking all of these data together, we hypothesized that plasma-activated seawater (PASW) may represent an eco-friendly option to depurate bivalve mollusks and control the biohazard risk linked to their microbiological contamination. As a first step to prove our hypothesis and given that experimental evidence on the efficacy of PASW to control foodborne pathogens is limited, we investigated the physico-chemical, antibacterial, and antiviral properties of three PASWs activated by different plasma exposures. Specifically, we determined the kinetic in vitro inactivation of *E. coli* O157, *Salmonella* spp., *V. parahemolyticus*, bacteriophages (MS2 and φ174), cultivable norovirus surrogates (MNV and TV), and HuNoV exposed to three different PASWs using traditional and advanced cell culture techniques to define and characterize bacterial and viral inactivation. RONS species were also determined. Ultimately, the most effective PASW was evaluated for its impact on cytotoxicity and mussel viability, simulating real-world applicability.

## 2. Materials and Methods

### 2.1. Production and Characterization of PASWs

Depurated seawater samples (SW) were collected from the clean seawater reservoir tanks of a commercial depuration center in Bari (Mare Gioioso SRL, Monopoli Bari, Italy). In particular, the depuration center collects seawater from the Adriatic Mediterranean Sea and filters and UV treats it to remove suspended particles and microbial contaminants before using it for mollusk depuration. The SW was collected in Pirex^®^ glassware and transferred to the laboratory.

An experimental jet–dielectric barrier discharge (DBD) plasma in a remote configuration was used for the treatments (Renasco S.R.L., Bari, Italy). Specifically, the plasma was ignited in atmospheric air used as the working gas (feed gas) and sustained within the reactor. The jet nozzle was placed at 5 cm from the liquid surface to promote direct contact between the plasma plume and the SW. Plasma was discharged from the plasma generator with an input voltage of 230 V, a total output of 800 watts, and a frequency of 30 kHz. Each batch consisted of 300 mL of SW in a 1000 mL beaker exposed to plasma treatment for 10 (PASW10), 20 (PASW20), or 30 (PASW30) min, obtaining three different PASWs. SW was used as a control. When higher volumes of PASW were needed (e.g., mussel viability), several batches of 300 mL were prepared, mixed together, and denoted as one batch.

Physico-chemical characterization of PASWs was carried out before and immediately after each plasma treatment. The pH and temperature were monitored using a pH meter (Hanna Instrument, Padova, Italy) and a digital thermometer probe (Fisherbrand™ Traceable™, Waltham, MA, USA), respectively. Electrical conductivity was measured using a portable electrical conductivity probe (Hanna Instrument, HI 8633, Italy). Semi-quantitative test strips were used for hydrogen peroxide (H_2_O_2_), nitrite (NO_2_^−^), and nitrate (NO_3_^−^) (QUANTOFIX, Macherey-Nagel, Milano, Italy) measurements. All inactivation experiments, including bacteria, phages, surrogates, and HuNoV, were carried out at a room temperature of 22 ± 2 °C.

### 2.2. Phage Inactivation by PASWs

F-specific (MS2) and somatic (φ174) bacteriophages were initially used to investigate the antiviral effect of PASWs. A double-layer plaque assay was used to quantify phage inactivation using the *E. coli* CECT 9198 and *E. coli* WG5 strains as host strains for wild-type MS2 bacteriophage DSM 13767 (DSMZ, Braunschweig, Germany) and φ174 (Bluephage, Barcelona, Spain), respectively [53].

Tryptone soy agar (TSA) (Oxoid, Thermofisher, Madrid, Spain) was used for the lower layers, and semisolid tryptone soy broth (sTSB) (Oxoid, Thermofisher, Madrid, Spain) with 0.5% bacteriological agar (Oxoid, Thermofisher, Madrid, Spain) and 0.04 M magnesium chloride was used for the upper layers. *E. coli* CECT 9198 and *E. coli* WG5 were initially grown in TSB (Oxoid, Thermofisher, Madrid, Spain) at 37 °C until the log phase was reached (optical density, OD = 0.3). Then, a 1 mL ten-fold serial dilution of phage sample was added to 5 mL sTSB along with 1 mL of *E. coli* host strain (OD = 0.3), mixed, and carefully plated on TSA. The plates were incubated overnight at 37 °C and the phage load was determined as plaque-forming units per mL (PFUs/mL). Phage decay was calculated as log_10_ S (N/N0), where N0 is the titer in the control sample and N is the titer in the plasma-treated one. Phage decay following 24 h exposure to PASW10, PASW20, and PASW30 was investigated for MS2 and φ174. Briefly, 100 µL of 7 log plaque-forming units (PFUs)/mL for MS2 or from 6 log PFUs/mL for φ174 were mixed with 900 µL of each PASW sample. Phage suspensions in phosphate-buffered solution (PBS) pH 7.4 and SW were included as controls.

### 2.3. Surrogate Inactivation by PASWs

MNV and TV were used as human norovirus surrogates to investigate kinetic inactivation due to exposure to PASWs. MNV (provided by Prof. H. W. Virgin, Washington University School of Medicine, USA) and TV (provided by Prof. Farkas, Louisiana State University, LA, USA) were propagated and assayed respectively in RAW 264.7 and LLC-MK2 (ATCC CCL-7) cells as previously described in [50,54]. Briefly, MNV and TV were cultured in Dulbecco’s modified Eagle medium (DMEM, Gibco Life Technologies, Madrid, Spain) supplemented with 10% fetal bovine serum (FBS) and Opti-MEM (Gibco Life Technologies) supplemented with 2% FBS.

Viral decay following exposure to PASW10, PASW20, and PASW30 was investigated for each surrogate by collecting samples at 0, 0.5, 1, 3.0, 12, and 24 h to determine their kinetical inactivation. Briefly, 100 µL of 5–6 log 50% tissue culture infectious dose (TCID_50_)/mL was mixed with 900 µL of each PASW sample. Viral suspensions in PBS pH 7.4, PBS pH 5.0, and SW were included as controls. Samples collected at selected time points were neutralized by 1:10 (*v*/*v*) dilution in cell culture media supplemented with 10% FBS. Viral titer was determined using the TCID_50_ assay inoculating 20 μL of 10-fold serial dilutions of MNV or TV prepared in PBS pH 7.4 on eight wells with 70–80% confluent monolayers in 96-well plates. After incubation for 1 h at 37 °C, 180 μL DMEM or Opti-MEM was added. After 2 to 5 days, cells showing cytopathic effect (CPE) were enumerated by visual inspection, and the titer of infectious viruses was determined using the Spearman–Karber method.

### 2.4. Human Norovirus Inactivation by PASWs

HuNoV GII.4 decay following 24 h exposure to PASW10, PASW20, and PASW30 was investigated using the HIE model. Three-dimensional (3D) HIE derived from human jejunal biopsy (J2 cell line, provided by Prof. Mary K. Estes, Baylor College of Medicine, Houston, TX) and differentiated monolayers were routinely maintained and produced as described by Carmona and Randazzo [55]. The HuNoV GII.4 Sydney [P16]-positive fecal sample (provided by Prof. Buesa, University of Valencia, Valencia, Spain) was de-identified, tested for infectivity in the HIE system, and used for inactivation experiments. The experimental design consisted of exposing 100 µL of 9 log genome copies (GC)/100 µL HuNoV GII.4 to 900 µL of each PASW sample for 24 h. HuNoV suspensions in PBS pH 7.4 and SW were included as controls. Then, each sample was neutralized by mixing 1:10 (*v*/*v*) with organoid differentiation medium (ODM) and infected in two sets of 96-well plates with 100% confluent 7–10 day-old differentiated HIE monolayers.

Undifferentiated 3D HIEs and differentiated monolayers were maintained and produced using commercial IntestiCult™ Organoid Medium Human media (STEMCELL Technologies Inc., Vancouver, CO, Canada) as already described [55]. Differentiated HIE monolayers were typically 100% confluent after five to seven days and used for HuNoV infections. Two sets of 96-well plates with 100% confluent differentiated HIE monolayers were inoculated in triplicate with 100 μL of each HuNoV sample diluted at 1:10 (*v*/*v*) in complete media without growth factors (CMGF-) supplemented with 500 μM sodium glycochenodeoxycholate. After 1 h incubation at 37 °C, the inoculum was removed, monolayers were washed twice with CMGF-, and 100 μL of ODM was added to each well. For each set of infections, one 96-well plate was immediately frozen at −80 °C (1 hpi) and the second plate was incubated at 37 °C and 5% CO_2_ for 48 h (48 hpi) and then frozen. Three technical replicates were included for each sample.

Finally, RT-qPCR was used to determine the amount of HuNoV RNA from HIE monolayers at 1 and 48 hpi to evaluate HuNoV replication. Briefly, viral RNA was extracted using the Maxwell^®^ RSC Instrument (Promega, Madrid, Spain) according to the manufacturer’s instructions. Then, it was detected in duplicate via TaqMan RT-qPCR using the RNA UltraSense One-Step quantitative RT-PCR system (Invitrogen, Madrid, Spain) on a LightCycler 480 instrument (Roche Diagnostics, Barcelona, Spain). The primers and probe included in the ISO 15216-1, 2017 were used for detecting HuNoV GII [39]. Ten-fold serial dilutions of synthetic gBlock gene fragments (IDT) were used to build an external standard curve, and HuNoV RNA was quantified as genome equivalents (HuNoV GII: y = −3.56x + 40.664, R = 0.997). Positive and negative amplification controls were included in each RT-qPCR run, along with the inhibitory amplification control consisting of a 10-fold diluted RNA for each sample.

### 2.5. Bacterial Inactivation by PASWs

The efficacy of PASW was investigated using *E. coli*, *Salmonella* spp., and V. parahaemolyticus. Specifically, *E. coli* O157 and *Salmonella* spp. strains were maintained in sterile glycerol solution at −80 °C in the laboratory collection. The V. parahaemolyticus 17802 reference strain was supplied by ATCC. *E. coli* O157 and *Salmonella* spp. were maintained in tryptone soy broth (TSB, Oxoid, ThermoFisher, Milano, Italy) and Rappaport–Vassiliadis medium (RV, Oxoid, ThermoFisher, Milano, Italy), respectively. V. parahemolyticus was grown in TSB medium supplemented with 3% NaCl. Maximum recovery diluent (MRD, Oxoid, ThermoFisher, Milano, Italy) medium was used to prepare ten-fold dilutions, and a plate count assay was used for bacterial titration. Specifically, *E. coli* O157 and *Salmonella* spp. were titrated after 24 h incubation in plate count agar (PCA, Oxoid, ThermoFisher, Milano, Italy) at 37 °C, while V. parahaemolyticus was titrated after 24 h incubation in PCA supplemented with 3% NaCl at 41 °C.

Bacterial decay following exposure to PASW30 was investigated for each bacterial species, and samples were collected at 0, 0.5, 1.5, 2.5, 3.5, and 24 h to determine kinetic inactivation. Briefly, 1 mL of 10^6^ colony-forming units (CFUs)/mL overnight bacterial suspension was added to 100 mL of PASWs. At each time point, 1 mL of sample was collected, serial dilutions were performed to stop the reaction, and bacterial counts were determined as previously detailed. Bacterial suspensions in PBS pH 7.4 and SW were included as controls.

### 2.6. HIE Cytotoxicity

The cytotoxicity effect of PASW10, PASW20, and PASW30 on intestinal epithelial cells obtained from the differentiation of 3D HIE was investigated. The HIE system was maintained as previously described [55]. Each PASW sample was tested undiluted and diluted at 1:10 (*v*/*v*) in CMGF-. HIE monolayers were inoculated with 100 µL of each sample and incubated at 37 °C in a 5% CO_2_ incubator for 1 h. Then, monolayers were washed twice with CMGF- and maintained at 37 °C and 5% CO_2_ for 48 h with ODM. The potential cytotoxicity effect was monitored by visual inspection of monolayers using optical microscopy for the duration of the experiment. Finally, HIE monolayers were infected with HuNoV, and viral replication was assessed as previously described to check for cell functionality after exposure to different PASWs.

### 2.7. Mussel Viability

The effect of PASW30 on live mussels was investigated. Mediterranean mussels (*Mytilus galloprovincialis*) harvested from an A area were purchased from a local market in Bari (Italy). Forty mussels of similar size were selected, excluding under- and oversized mollusks, and transferred into a plastic tank filled with 20 L SW as a control or PASW30 for 24 h. The seawater in the feeding tank was aerated continuously during the experiment to preserve the physiological state of the mussels. The vitality of mussels was monitored every 3 h, checking the opening of their valves and their responsiveness to external stimuli.

### 2.8. Statistical Analyses

All data were compiled from three independent experiments with three technical replicates each. Statistical analyses of data were computed using GraphPad Prism software version 8. A one-way analysis of variance (ANOVA) was performed with a subsequent Tukey’s multiple comparison test by assuming a normal data distribution. The statistical results of measurements are shown as the mean value ± standard deviation (SD). *p*-values < 0.05 were considered statistically significant.

## 3. Results

### 3.1. Characterization of PASWs

Physico-chemical characterization of PASW10, PASW20, and PASW30 was carried out to determine the main RONS families generated in SW following the three plasma treatments (Table 1). The concentration of nitrites (NO_2_^−^) increased according to the duration of the plasma treatments, resulting in PASW10 < PASW20 < PASW30. On the contrary, the content of nitrates (NO_3_^−^) and peroxides (H_2_O_2_) was not directly correlated with the duration of the plasma treatment applied to the seawater. However, acidification of seawater samples was observed concomitant to the duration of the plasma treatment, as the pH decreased from 8.13 in SW to 7.95 in PASW10 and 5.00 in PASW30. Electrical conductivity resulted in 47.5 ± 1.23 μS/cm on average with no significant differences among the tested PASWs (*p* > 0.05).

### 3.2. Phage Inactivation by PASWs

**Figure 1:**
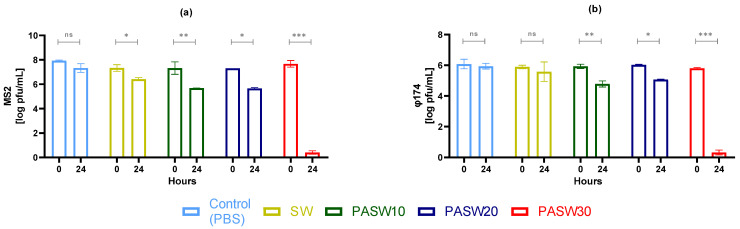
Inactivation of phages in plasma-activated seawaters (PASW10, PASW20, and PASW30). Phosphate-buffered solution (PBS) and seawater (SW) served as controls. (**a**) MS2 phage; (**b**) φ174 phage. Error bars indicate SDs; asterisks indicate significant differences between time points for each sample (*n* = 9): ns, no significant; * *p* < 0.05; ** *p* < 0.01; *** *p* < 0.001.

MS2 and φ174 phages were exposed to PASW10, PASW20, and PASW30 for 24 h (Figure 1). PBS pH 7.4 and SW served as controls. The results of three independent experiments showed comparable inactivation patterns for both phages. In particular, exposure to PASW10 and PASW20 inactivated both phages by 0.95 to 1.65 log PFUs/mL. In contrast, PASW30 inactivated MS2 and φ174 phages to undetectable levels within 24 h.

### 3.3. Kinetic Inactivation of HuNoV Surrogates

**Figure 2:**
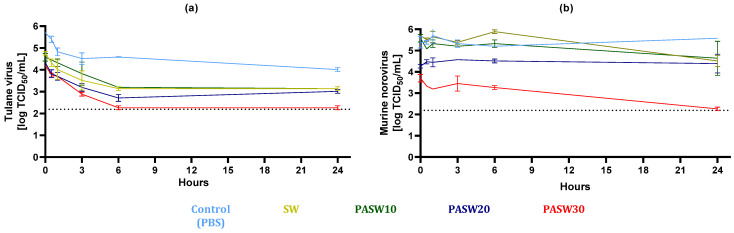
Kinetic inactivation of TV and MNV in plasma-activated seawaters (PASW10, PASW20, PASW30). Phosphate-buffered solution (PBS pH 7.4) and seawater (SW) served as controls. (**a**) TV; and (**b**) MNV.

The kinetic inactivation of TV and MNV, serving as surrogates for HuNoV, was investigated, and the results are presented in Figure 2. Specifically, viral suspensions were prepared in PBS pH 7.4, PBS pH 5.0, SW pH 8.2, SW pH 5.0, PASW10, PASW20, and PASW30, and viral titers were monitored at 0 (control), 0.5, 1, 3, 6, or 24 h. In general, the inactivation effect of PASWs on viruses was associated with the intensity of the plasma treatment (PASW10 < PASW20 < PASW30). No statistical differences were noticed among PBS pH 7.4, PBS pH 5.0, SW pH 8.2, and pH 5.0 used as experimental controls for either MNV or TV.

The titer of TV gradually decreased during 24 h in all samples, including in the PBS and SW controls. PASW10 showed comparable inactivation kinetics to those recorded for SW, indicating a limited effect on TV. However, the inactivation measured in the PASW20 and PASW30 samples was significantly higher, resulting in reductions of 2.99 and 3.44 log TCID_50_/mL after 6 h compared to PBS. This indicates that PASW30 inactivated TV after 6h to non-detectable levels.

MNV ended up being kinetically more stable than TV over time (Figure 2b). However, significant MNV decay was observed in PASW30, in which complete inactivation was reached within 24 h. This implied a 3.13 log TCID_50_/mL reduction with respect to the PBS pH 7.4 control. PASW30 completely inactivated the TV and MNV titers within 24 h.

### 3.4. Inactivation of HuNoV

**Figure 3:**
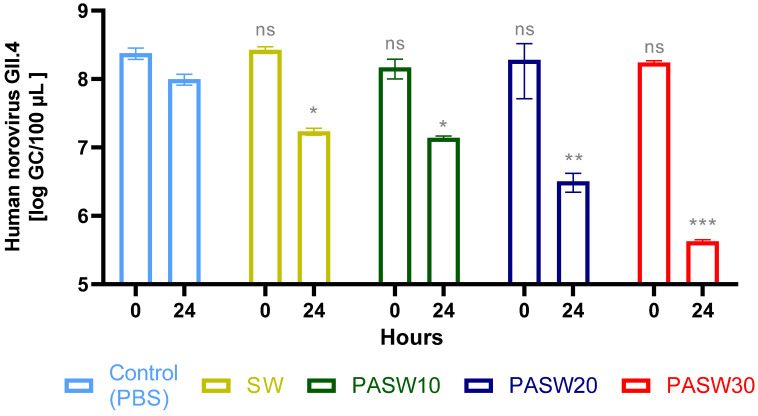
Replication of HuNoV following exposure for 24 h to PASW10, PASW20, and PASW30. Phosphate-buffered solution (PBS pH 7.4) and seawater (SW) served as controls. Error bars indicate SDs; asterisks indicate a significant difference between the samples and the untreated control (PBS) at analogous time exposure points: ns, no significant; * *p* < 0.05; ** *p* < 0.01; *** *p* < 0.001. The data are shown as the mean values ± standard errors of three independent experiments (*n* = 9).

HuNoV GII.4 was mixed with PBS pH 7.4, SW, PASW10, PASW20, and PASW30, and samples were collected at 0 and 24 h time points. These samples were used to infect the HIE monolayers. HuNoV RNA was extracted and quantified from cell supernatant at 0 and 48 hpi to calculate viral replication. The initial HuNoV concentration was 6.1 ± 0.1 log GC/100µL. All samples at time 0 showed replication at a comparable extent to the PBS control, reaching a titer of 8.4 log GC/100 µL (Figure 3). Samples collected after 24 h in PBS, SW, PASW10, M-PASW, and PASW30 showed different replication rates. Specifically, HuNoV replication decreased by 0.76 and 0.86 log GC/100 µL in SW and PASW10. Inactivation of 1.49 log GC/100 µL was observed in PASW20, and the complete prevention of HuNoV replication was detected in PASW30 (2.37 log GC/100 µL reduction).

### 3.5. Kinetic Bacterial Inactivation

**Figure 4:**
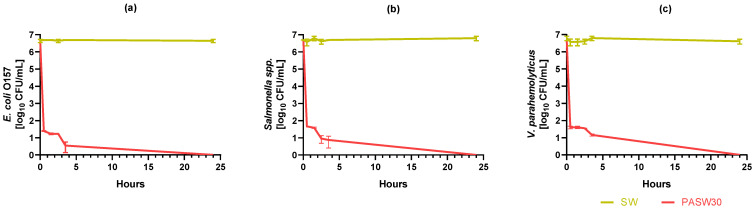
Kinetic bacterial inactivation in plasma-activated seawater (PASW30). Untreated seawater (SW) served as a control. (**a**) *E. coli* O157; (**b**) *Salmonella* spp.; (**c**) *V. parahemolyticus*.

The kinetic inactivation of three bacterial pathogens exposed to PASW30 for 24 h is shown in Figure 4. The average initial *E. coli* O157, *Salmonella* spp., and *V. parahemolyticus* load was 6.63 ± 0.09, 6.69 ± 0.01, and 6.79 ± 0.12 log CFUs/mL, respectively. No statistically significant reductions in bacterial titers were detected during 24 h in SW. In PASW30, the initial load was statistically reduced by >5 log after 0.5 h. In particular, *E. coli* O157, *Salmonella* spp., and *V. parahemolyticus* were reduced to 1.39 ± 0.01, 1.66 ± 0.00, and 1.60 ± 0.08 log CFU/mL, respectively. Residual bacterial counts steadily decreased during the following 3.5 h monitoring period, and complete inactivation was reached within 24 h for all three tested bacteria.

### 3.6. HIE Cytotoxicity

Given the presence of different concentrations of RONS due to the intensity of each plasma treatment in seawater, PASWs undiluted and diluted at 1:10 were tested on HIE monolayers for 1 h exposure. When tested undiluted, PASWs affected the monolayer integrity, and HuNoV infections were not conducted. On the contrary, no morphological changes in the monolayer structure and uniformity were observed during the visual monitoring of HIE exposed to PASW10, PASW20, and PASW30 diluted 1:10 in CGMF-. For all tested diluted PASWs, the HuNoV replication rates (8.22 ± 0.82 log GC/100 µL) showed no statistical differences (*p* > 0.05) compared to the PBS control (8.15 ± 0.75 log GC/100 µL).

### 3.7. Mussel Viability

Given the promising antibacterial and antiviral activity of PASW30 and its significant concentration of RONS, its effect on mussel viability was investigated in vivo. A total of 40 mussels for each treatment condition (PASW30 and SW) were used in the experiment owing to the need to monitor mortality within 24 h. All mussels in the depuration treatments were observed to open their valves after being left undisturbed, demonstrating their filter-feeding activity. However, a total of four mussels perished after 24 h in SW, possibly due to weakness and being in an unfamiliar environment. In PASW30, all mussels survived and were found to be responsive to external stimuli until 6 h of treatment, even though a total of seven mollusks died at 24 h.

## 4. Discussion

Current European regulations and commercial depuration processes fail to fully guarantee the microbiological safety of shellfish [12,25,26,27,38]. Despite there being several studies confirming the effectiveness of PAW in inactivating bacterial pathogens, our study reports novel data on the production of PASW, its efficacy in controlling relevant foodborne viruses and bacteria, and its potential application as a strategy for shellfish depuration.

Our investigation focused on the most relevant pathogens involved in shellfish outbreaks, such as HuNoV and *V. parahemolyticus*, along with additional indicators of microbiological safety (e.g., *E. coli* and phages). Specifically, the DBD plasma device activating SW for 30 min resulted in PASW30, which was able to completely inactivate *E. coli* O157, *Salmonella* spp. and *V. parahemolyticus*, MS2 and φ174 phages, and TV, MNV, and HuNoV within 24 h.

Notably, our experimental design included innovative techniques (e.g., an HIE system) able to robustly inform us about HuNoV infectivity and inactivation. Previous studies inferred HuNoV decay using molecular methods such as RT-qPCR or viability RT-qPCR, which have not overcome the limitation of the sound correlation of infectivity and molecular assays [56], including for plasma-mediated inactivation [57]. Alternatively, surrogates have been employed, even though their use has been questioned and resulting patterns still need to be correlated with HuNoVs infectivity. Our results indicate that MNV is more stable than TV over time when treated with PASWs (Figure 2). Comparing surrogates with HuNoV GII.4, the viral response to the diverse tested PASWs was qualitatively comparable, with PASW30 being able to completely prevent viral replication in the cell models. In addition, complete inactivation of high titer MS2 and ϕ174 phages (6–8 log PFUs/mL) was observed within 24 h for PASW30 but not for PASW10 or PASW20, providing further evidence of the effectiveness of the treatment in controlling viral contamination.

PASW30 was demonstrated to be effective in inactivating bacterial pathogens, namely *E. coli* O157, *Salmonella* spp., and *V. parahemolyticus*. Significant reduction (>5 log) was achieved within the first 30 min of exposure, and complete inactivation was reached within 24 h for all tested bacteria. Comparing the kinetic datasets, bacteria and TV showed consistent inactivation patterns, even though bacterial decay was faster than viral one.

Taking these data together, microbial inactivation was associated with the intensity of the plasma treatment (with it being PASW10 < PASW20 < PASW30), and it was time dependent. The physical–chemical characterization of PASWs indicated the production of different concentrations of RONS generated in SW following plasma activation (Table 1). Specifically, nitrites (NO_2_^−^) increased according to the duration of the plasma treatments, while the content of nitrates (NO_3_^−^), and peroxides (H_2_O_2_) was not proportional. Thus, our data suggest that nitrites may be the primary agent responsible for viral inactivation.

A comprehensive comparison of our data with the available literature is difficult as wide differences have been found related to plasma technology (e.g., plasma sources and gas type), type of water (e.g., water, salt water, simulated seawater, and seawater), tested microorganisms, and inactivation conditions [24].

Only a few reports have investigated plasma-activated salt water or seawater, and its use for microbial inactivation [58,59,60,61,62,63]. Salt concentration affects electrical conductivity, and thus, it is directly related to the number of ions generated in solution during the plasma activation process. This contributes to the production of RONS and other antimicrobial compounds (e.g., singlet oxygen) responsible for microbial inactivation [11,63,64].

Focusing on PASWs, we successfully plasma-activated seawater using actual seawater, with demonstrated antimicrobial activity (Figure 1, Figure 2, Figure 3 and Figure 4) and characterized by concentrations of NO_3_^−^ similar to a previous study carried out using simulated seawater [63]. Importantly, the NO_3_^−^ concentrations of PASW30 (310 mg/mL) (Table 1) fall within the range reported to avoid adverse effects on clams’ and oysters’ viability (320 mg/mL) [65].

Our viability assays on mussels provide additional evidence that PASW within the aforementioned limit has a limited impact on mussels’ viability. Similar considerations apply to pH as well. Indeed, the decrease in pH in PAW and PASW is attributed to the formation of inorganic acids (e.g., HNO_2_ and HNO_3_) that contribute to altering microbial and viral stability [66,67,68,69,70,71], while they can also affect shellfish viability [72]. Thus, PASW is diluted or buffered to prevent premature shellfish mortality [62,63]. However, the pH of PASW tested effectively in our study (PASW30 pH 5) is significantly different from pH < 3 reported previously [57,67]. This discrepancy is likely due to the diverse composition of seawater samples, with natural seawater carrying unidentified elements with buffering capacity (e.g., microalgae, organic matter, trace elements, or minerals) not included in the simulated seawater used by other authors [62,63].

Regarding antimicrobial activity, the results of this study were compared to previous studies using PAW, plasma-activated salt water, or simulated PASW since there are no reports on PASW. Campbell and colleagues first reported the reduction of ~3 log CFUs/mL *E. coli* treated with simulated PASW for 5 min [63]. The authors also reported that *E. coli* reductions were higher in PAW than in PASW produced at the same plasma exposure time, supporting the relevance of the water type in plasma activation as previously discussed. Baek and colleagues observed a reduction > 4 log for *E. coli* O157:H7 after 5 min of exposure to plasma-activated salt water (0.9% NaCl, *w*/*v*) [58]. Overall, these results are in accordance with the data obtained in our study where *E. coli* O157 exposed to PASW30 was reduced by >5 log after 30 min (Figure 4a). Yun reported the reduction of >3 log of three different *Vibrio* strains after 3 h PAW treatment. In our study, we achieved >5 log *V. parahemolyticus* inactivation after 0.5 h of treatment with PASW30 (Figure 4c), as well as for *Salmonella* spp. In contrast, other authors have reported the even faster inactivation of >5 log *Salmonella enterica* exposed to PAW within 1 min [73]. As we investigated one isolate per studied species, future works should evaluate the effect of PASWs against different strains or isolates of the same species to provide a comprehensive overview of intraspecies sensitivity to this technology.

A comprehensive and quantitative comparison of the viral inactivation measurements of the present study cannot be provided because of the diverse models adopted (e.g., phages, surrogates, and HIE), the different cell systems used, and techniques applied for viral titration (e.g., plaque count vs. TCID_50_ vs. fold RNA increase upon replication), as well as for the experimental design adopted (kinetic vs. endpoint measurements). Still, our dataset provides novel experimental evidence of viral inactivation in SW due to plasma treatments. Indeed, while reports on viral inactivation of PASW, either salt or simulated SW, are missing, some information is available on viral inactivation using PAW as there is an increasing research interest in the field [74]. For instance, Guo showed that PAW reduced MS2 and ϕ174 phages by >4 log after 4 h of treatment [11]. Furthermore, Wu and colleagues [75] demonstrated that MS2 inactivation was dependent on gas carriers and power levels and achieved it within 3 min under the tested conditions. Enveloped, single-stranded RNA animal viruses have also been tested in water solutions. Interestingly, Newcastle disease virus was completely inactivated after 30 min either in water or salt (0.9% NaCl) water solutions [60]. A significant 4.3 log TCID_50_/100 μL reduction in Feline calicivirus (FCV), acting as a surrogate for human norovirus, was reported after 5 min treatment in sterile water solution, even though weaker reductions of 0.9–2 log TCID_50_/100 μL were recorded in buffered solution [17]. In addition, Pepper mild mottle virus (PMMV) and Tobacco mosaic virus (TMV), non-enveloped plant viruses, have been successfully inactivated by plasma in water, of interest for irrigation water decontamination [76,77].

This study expands the knowledge on the inactivation of the main viral foodborne pathogen, namely HuNoV, using an infectivity assay to assess the efficacy of plasma technology in seawater, which could be exploited in further investigations. Moreover, our data showed the infectivity of HuNoV GII.4 and its surrogates after 24 h in SW. Although the HuNoV, TV, and MNV concentrations were slightly reduced, their infectivity demonstrated after 24 h in SW confirms the experimental evidence on the risk of viral transmissibility through the water cycle [49,78,79].

The mechanism of viral inactivation was not investigated in this study. However, several studies have reported that plasma-mediated virus inactivation is caused by RONS that induce the perforation of viral capsid and a reduction in virus replication [11,71,77,80]. On the other hand, the effect and synergy of hydrogen peroxide in the inactivation process of plasma treatments is not yet fully elucidated [11,81,82]. Besides the resistance of enteric viruses to slight acid conditions (e.g., pH 4–5) [70], experimentally confirmed in the present study as well, it cannot be excluded that the pH may exert an additive antiviral effect along with RONS. Thus, it would be worth deeply investigating these viral inactivation mechanisms in salt water solutions in future studies.

Our study provides evidence to exclude the potential adverse effects of the tested PASW on biological organisms. Indeed, we tested the potential cytotoxicity of PASW on cells using HIE. Diluted 1:1 PASWs were assessed as nontoxic as no structural damage of monolayers was observed, and cell functionality was preserved as HuNov replication was demonstrated to be supported. Previous studies used HepG2 cell lines or live, immunodeficient mice for similar purposes [76,83]. Besides the different characteristics of activated waters and the biological models used for testing, these studies corroborate the need for assessing the safety of PAW/PASW for its safe use and consumption.

Finally, we tested the viability of mussels in PASW30 within 24 h as the first step in employing PASWs in shellfish depuration systems. Interestingly, Campell and colleagues investigated the effective depuration of *E. coli* in bioaccumulated oysters and depurated in PASW for 24 h [62]. The authors reported that 24 h PASW depuration of bioaccumulated oysters reduced total coliforms and *E. coli* by 3.9 and 3.4 logs, respectively, without any negative effect on mollusk viability or quality. Thus, the use of bioaccumulated shellfish with phages or norovirus surrogates and depurated with PASW could represent the next research step to define effective operational procedures of depuration strategies for different bivalve mollusk species.

## 5. Conclusions

This study proves PASWs to be an efficient technology to inactivate *E. coli* O157, *Salmonella* spp., *V. parahemolyticus*, bacteriophages (MS2 and ϕ174), cultivable norovirus surrogates (MNV and TV), and HuNoV. In particular, PASW activated for 30 min completely inactivated the studied pathogens within 24 h, while preventing cytotoxicity effects and preserving mussel viability. Future studies should validate this antiviral and antibacterial effect of PASWs in bioaccumulated shellfish in a simulated depuration process.

Taking all of these results together, this research shows that PASW can be a suitable option to depurate bivalve mollusks and control the biohazard risks linked to microbiological contamination, either viral or bacterial, due to its high efficiency, short treatment period, sustainability, and cost-effectiveness.

## Figures and Tables

**Table 1:** Physico-chemical characterization of PASWs.

PASW	NO_2_^−^ (mg/L)	NO_3_^−^ (mg/L)	H_2_O_2_ (mg/L)	pH
SW	0	0	0	8.13 ± 0.31 ^b^
PASW10	5 ± 0.5 ^b^	25 ± 1 ^a^	1 ± 0.2 ^b^	7.95 ± 0.11 ^b^
PASW20	0.5 ± 0.5 ^a^	298 ± 3 ^b^	5 ± 0.3 ^c^	7.73 ± 0.17 ^b^
PASW30	100 ± 0.5 ^c^	310 ± 10 ^b^	0.5 ± 0.1 ^a^	5.00 ± 0.22 ^a^

Different letters indicate significant differences among PASWs (*p* < 0.05). The data are shown as the mean values ± standard errors of three independent physico-chemical determinations.

## Data Availability

The original contributions presented in the study are included in the article, further inquiries can be directed to the corresponding authors.

## References

[1] Šimončicová J., Kryštofová S., Medvecká V., Ďurišová K., Kaliňáková B. (2019). Technical Applications of Plasma Treatments: Current State and Perspectives. Appl. Microbiol. Biotechnol..

[2] Bourke P., Ziuzina D., Boehm D., Cullen P.J., Keener K. (2018). The Potential of Cold Plasma for Safe and Sustainable Food Production. Trends Biotechnol..

[3] Zhou X., Cai D., Xiao S., Ning M., Zhou R., Zhang S., Chen X., Ostrikov K., Dai X. (2020). In Vivo Pen: A Novel Plasma Source for In Vivo Cancer Treatment. J. Cancer.

[4] Wang Q., Salvi D. (2021). Recent Progress in the Application of Plasma-Activated Water (PAW) for Food Decontamination. Curr. Opin. Food Sci..

[5] Oh Y.J., Song A.Y., Min S.C. (2017). Inhibition of Salmonella Typhimurium on Radish Sprouts Using Nitrogen-Cold Plasma. Int. J. Food Microbiol..

[6] Fernández A., Noriega E., Thompson A. (2013). Inactivation of Salmonella Enterica Serovar Typhimurium on Fresh Produce by Cold Atmospheric Gas Plasma Technology. Food Microbiol..

[7] Choi S., Puligundla P., Mok C. (2016). Corona Discharge Plasma Jet for Inactivation of *Escherichia coli* O157:H7 and Listeria Monocytogenes on Inoculated Pork and Its Impact on Meat Quality Attributes. Ann. Microbiol..

[8] Baier M., Görgen M., Ehlbeck J., Knorr D., Herppich W.B., Schlüter O. (2014). Non-Thermal Atmospheric Pressure Plasma: Screening for Gentle Process Conditions and Antibacterial Efficiency on Perishable Fresh Produce. Innov. Food Sci. Emerg. Technol..

[9] Hertwig C., Reineke K., Ehlbeck J., Knorr D., Schlüter O. (2015). Decontamination of Whole Black Pepper Using Different Cold Atmospheric Pressure Plasma Applications. Food Control.

[10] Thirumdas R., Kothakota A., Annapure U., Siliveru K., Blundell R., Gatt R., Valdramidis V.P. (2018). Plasma Activated Water (PAW): Chemistry, Physico-Chemical Properties, Applications in Food and Agriculture. Trends Food Sci. Technol..

[11] Guo L., Xu R., Gou L., Liu Z., Zhao Y., Liu D., Zhang L., Chen H., Kong M.G. (2018). Mechanism of Virus Inactivation by Cold Atmospheric-Pressure Plasma and Plasma-Activated Water. Appl. Environ. Microbiol..

[12] Aboubakr H.A., Williams P., Gangal U., Youssef M.M., El-Sohaimy S.A.A., Bruggeman P.J., Goyal S.M. (2015). Virucidal Effect of Cold Atmospheric Gaseous Plasma on Feline Calicivirus, a Surrogate for Human Norovirus. Appl. Environ. Microbiol..

[13] Baert L., Debevere J., Uyttendaele M. (2009). The Efficacy of Preservation Methods to Inactivate Foodborne Viruses. Int. J. Food Microbiol..

[14] Ahlfeld B., Li Y., Boulaaba A., Binder A., Schotte U., Zimmermann J.L., Morfill G., Klein G. (2015). Inactivation of a Foodborne Norovirus Outbreak Strain with Nonthermal Atmospheric Pressure Plasma. mBio.

[15] Bae S.-C., Park S.Y., Choe W., Ha S.-D. (2015). Inactivation of Murine Norovirus-1 and Hepatitis a Virus on Fresh Meats by Atmospheric Pressure Plasma Jets. Food Res. Int..

[16] Lacombe A., Niemira B.A., Gurtler J.B., Sites J., Boyd G., Kingsley D.H., Li X., Chen H. (2017). Nonthermal Inactivation of Norovirus Surrogates on Blueberries Using Atmospheric Cold Plasma. Food Microbiol..

[17] Nayak G., Aboubakr H.A., Goyal S.M., Bruggeman P.J. (2018). Reactive Species Responsible for the Inactivation of Feline Calicivirus by a Two-dimensional Array of Integrated Coaxial Microhollow Dielectric Barrier Discharges in Air. Plasma Process. Polym..

[18] Herianto S., Shih M.-K., Lin C.-M., Hung Y.-C., Hsieh C.-W., Wu J.-S., Chen M.-H., Chen H.-L., Hou C.-Y. (2022). The Effects of Glazing with Plasma-Activated Water Generated by a Piezoelectric Direct Discharge Plasma System on Whiteleg Shrimp (*Litopenaeus vannamei*). LWT.

[19] Patange A., Lu P., Boehm D., Cullen P.J., Bourke P. (2019). Efficacy of Cold Plasma Functionalised Water for Improving Microbiological Safety of Fresh Produce and Wash Water Recycling. Food Microbiol..

[20] Andrasch M., Stachowiak J., Schlüter O., Schnabel U., Ehlbeck J. (2017). Scale-up to Pilot Plant Dimensions of Plasma Processed Water Generation for Fresh-Cut Lettuce Treatment. Food Packag. Shelf Life.

[21] Wong K.S., Lim W.T.H., Ooi C.W., Yeo L.Y., Tan M.K. (2020). In Situ Generation of Plasma-Activated Aerosols via Surface Acoustic Wave Nebulization for Portable Spray-Based Surface Bacterial Inactivation. Lab Chip.

[22] de Castro Medeiros L., de Alencar F.L.S., Navoni J.A., de Araujo A.L.C., do Amaral V.S. (2019). Toxicological Aspects of Trihalomethanes: A Systematic Review. Environ. Sci. Pollut. Res..

[23] Jenns K., Sassi H.P., Zhou R., Cullen P.J., Carter D., Mai-Prochnow A. (2022). Inactivation of Foodborne Viruses: Opportunities for Cold Atmospheric Plasma. Trends Food Sci. Technol..

[24] Rahman M., Hasan M.S., Islam R., Rana R., Sayem A.S.M., Sad M.A.A., Matin A., Raposo A., Zandonadi R.P., Han H. (2022). Plasma-Activated Water for Food Safety and Quality: A Review of Recent Developments. Int. J. Environ. Res. Public Health.

[25] Oliveira J., Cunha A., Castilho F., Romalde J.L., Pereira M.J. (2011). Microbial Contamination and Purification of Bivalve Shellfish: Crucial Aspects in Monitoring and Future Perspectives—A Mini-Review. Food Control.

[26] RASFF Window. https://webgate.ec.europa.eu/rasff-window/screen/search?searchQueries=eyJkYXRlIjp7InN0YXJ0UmFuZ2UiOiIyMDIyLTEyLTMxVDIzOjAwOjAwLjAwMFoiLCJlbmRSYW5nZSI6IjIwMjMtMTItMzBUMjM6MDA6MDAuMDAwWiJ9LCJub3RpZmljYXRpb25TdGF0dXMiOnsibm90aWZpY2F0aW9uU3RhdHVzIjpbWzFdXX0sInByb2R1Y3QiOnsicHJvZHVjdENhdGVnb3J5IjpbWzE4NDMyXV19LCJyaXNrIjp7ImhhemFyZENhdGVnb3J5IjpbWzIxODAxXV19fQ%3D%3D.

[27] European Food Safety Authority (EFSA) (2019). Analysis of the European Baseline Survey of Norovirus in Oysters. EFSA J..

[28] Baker-Austin C., Oliver J.D., Alam M., Ali A., Waldor M.K., Qadri F., Martinez-Urtaza J. (2018). *Vibrio* spp. Infections. Nat. Rev. Dis. Primers.

[29] Mancini M.E., Alessiani A., Donatiello A., Didonna A., D’Attoli L., Faleo S., Occhiochiuso G., Carella F., Di Taranto P., Pace L. (2023). Systematic Survey of *Vibrio* spp. and *Salmonella* spp. in Bivalve Shellfish in Apulia Region (Italy): Prevalence and Antimicrobial Resistance. Microorganisms.

[30] Mok J.S., Ryu A., Kwon J.Y., Kim B., Park K. (2019). Distribution of *Vibrio* Species Isolated from Bivalves and Bivalve Culture Environments along the Gyeongnam Coast in Korea: Virulence and Antimicrobial Resistance of *Vibrio parahaemolyticus* Isolates. Food Control.

[31] Directive 2003/99/EC of the European Parliament and of the Council of 17 November 2003 on the Monitoring of Zoonoses and Zoonotic Agents, Amending Council Decision 90/424/EEC and Repealing Council Directive 92/117/EEC. http://data.europa.eu/eli/dir/2003/99/oj.

[32] European Food Safety Authority, European Centre for Disease Prevention and Control (2022). The European Union One Health 2021 Zoonoses Report. EFSA J..

[33] Commission Regulation (EC) No 1441/2007 of 5 December 2007 Amending Regulation (EC) No 2073/2005 on Microbiological Criteria for Foodstuffs (Text with EEA Relevance). http://data.europa.eu/eli/reg/2007/1441/oj.

[34] European Food Safety Authority, European Centre for Disease Prevention and Control (2023). The European Union One Health 2022 Zoonoses Report. EFSA J..

[35] Pavoni E., Bertasi B., Galuppini E., Mangeri L., Meletti F., Tilola M., Carta V., Todeschi S., Losio M.-N. (2022). Detection of Hepatitis a Virus and Norovirus in Different Food Categories: A 6-Year Survey in Italy. Food Environ. Virol..

[36] Commission Implementing Regulation (EU) 2019/627 of 15 March 2019 Laying Down Uniform Practical Arrangements for the Performance of Official Controls on Products of Animal Origin Intended for Human Consumption in Accordance with Regulation (EU) 2017/625 of the European Parliament and of the Council and Amending Commission Regulation (EC) No 2074/2005 as Regards Official Controls (Text with EEA Relevance). http://data.europa.eu/eli/reg_impl/2019/627/oj.

[37] Le Mennec C., Parnaudeau S., Rumebe M., Le Saux J.C., Piquet J.C., Le Guyader S.F. (2017). Follow-Up of Norovirus Contamination in an Oyster Production Area Linked to Repeated Outbreaks. Food Environ. Virol..

[38] McLeod C., Polo D., Le Saux J., Le Guyader F.S. (2017). Depuration and Relaying: A Review on Potential Removal of Norovirus from Oysters. Compr. Rev. Food Sci. Food Saf..

[39] (2017). Microbiology of the Food Chain—Horizontal Method for Determination of Hepatitis a Virus and Norovirus Using Real-Time RT-PCR—Part 1: Method for Quantification.

[40] Estes M.K., Ettayebi K., Tenge V.R., Murakami K., Karandikar U., Lin S.C., Ayyar B.V., Cortes-Penfield N.W., Haga K., Neill F.H. (2019). Human Norovirus Cultivation in Nontransformed Stem Cell-Derived Human Intestinal Enteroid Cultures: Success and Challenges. Viruses.

[41] Kamarasu P., Hsu H.-Y., Moore M.D. (2018). Research Progress in Viral Inactivation Utilizing Human Norovirus Surrogates. Front. Sustain. Food Syst..

[42] Cromeans T., Park G.W., Costantini V., Lee D., Wang Q., Farkas T., Lee A., Vinjé J. (2014). Comprehensive Comparison of Cultivable Norovirus Surrogates in Response to Different Inactivation and Disinfection Treatments. Appl. Environ. Microbiol..

[43] Richards G.P. (2012). Critical Review of Norovirus Surrogates in Food Safety Research: Rationale for Considering Volunteer Studies. Food Environ. Virol..

[44] Blondin-Brosseau M., Harlow J., Doctor T., Nasheri N. (2021). Examining the Persistence of Human Coronavirus 229E on Fresh Produce. Food Microbiol..

[45] Danso D., Chow J., Streita W.R. (2019). Plastics: Environmental and Biotechnological Perspectives on Microbial Degradation. Appl. Environ. Microbiol..

[46] Ettayebi K., Crawford S.E., Murakami K., Broughman J.R., Karandikar U., Tenge V.R., Neill F.H., Blutt S.E., Zeng X.L., Qu L. (2016). Replication of Human Noroviruses in Stem Cell-Derived Human Enteroids. Science.

[47] Costantini V., Morantz E.K., Browne H., Ettayebi K., Zeng X.L., Atmar R.L., Estes M.K., Vinjé J. (2018). Human Norovirus Replication in Human Intestinal Enteroids as Model to Evaluate Virus Inactivation. Emerg. Infect. Dis..

[48] Escudero-Abarca B.I., Goulter R.M., Arbogast J.W., Leslie R.A., Green K., Jaykus L.A. (2020). Efficacy of Alcohol-Based Hand Sanitizers against Human Norovirus Using RNase-RT-QPCR with Validation by Human Intestinal Enteroid Replication. Lett. Appl. Microbiol..

[49] Desdouits M., Polo D., Le Mennec C., Strubbia S., Zeng X.L., Ettayebi K., Atmar R.L., Estes M.K., Le Guyader F.S. (2022). Use of Human Intestinal Enteroids to Evaluate Persistence of Infectious Human Norovirus in Seawater. Emerg. Infect. Dis..

[50] Randazzo W., Costantini V., Morantz E.K., Vinjé J. (2020). Human Intestinal Enteroids to Evaluate Human Norovirus GII.4 Inactivation by Aged-Green Tea. Front. Microbiol..

[51] Falcó I., Randazzo W., Pérez A., Martínez A., Rodrigo D., Sánchez G. (2023). High Pressure Treatment and Green Tea Extract Synergistically Control Enteric Virus Contamination in Beverages. Food Control.

[52] Kulawik P., Kumar Tiwari B. (2019). Recent Advancements in the Application of Non-Thermal Plasma Technology for the Seafood Industry. Crit. Rev. Food Sci. Nutr..

[53] Abedon S.T., Yin J. (2009). Bacteriophage Plaques: Theory and Analysis. Bacteriophages.

[54] Falcó I., Randazzo W., Rodríguez-Díaz J., Gozalbo-Rovira R., Luque D., Aznar R., Sánchez G. (2019). Antiviral Activity of Aged Green Tea Extract in Model Food Systems and under Gastric Conditions. Int. J. Food Microbiol..

[55] Carmona N., Randazzo W. (2023). Passaging human intestinal organoids and monolayers set up. Protoc. Exch..

[56] Wales S.Q., Pandiscia A., Kulka M., Sánchez G., Randazzo W. (2024). Challenges for Estimating Human Norovirus Infectivity by Viability RT-QPCR as Compared to Replication in Human Intestinal Enteroids. Int. J. Food Microbiol..

[57] Aboubakr H.A., Sampedro Parra F., Collins J., Bruggeman P., Goyal S.M. (2020). Ìn Situ Inactivation of Human Norovirus GII.4 by Cold Plasma: Ethidium Monoazide (EMA)-Coupled RT-QPCR Underestimates Virus Reduction and Fecal Material Suppresses Inactivation. Food Microbiol..

[58] Baek K.H., Yong H.I., Yoo J.H., Kim J.W., Byeon Y.S., Lim J., Yoon S.Y., Ryu S., Jo C. (2020). Antimicrobial Effects and Mechanism of Plasma Activated Fine Droplets Produced from Arc Discharge Plasma on Planktonic *Listeria monocytogenes* and *Escherichia coli* O157:H7. J. Phys. D Appl. Phys..

[59] Oehmigen K., Winter J., Hähnel M., Wilke C., Brandenburg R., Weltmann K., von Woedtke T. (2011). Estimation of Possible Mechanisms of *Escherichia coli* Inactivation by Plasma Treated Sodium Chloride Solution. Plasma Process. Polym..

[60] Su X., Tian Y., Zhou H., Li Y., Zhang Z., Jiang B., Yang B., Zhang J., Fang J. (2018). Inactivation Efficacy of Nonthermal Plasma-Activated Solutions against Newcastle Disease Virus. Appl. Environ. Microbiol..

[61] Wang Q., Salvi D. (2021). Evaluation of Plasma-Activated Water (PAW) as a Novel Disinfectant: Effectiveness on Escherichia Coli and Listeria Innocua, Physicochemical Properties, and Storage Stability. LWT.

[62] Campbell V.M., Hall S., Salvi D. (2023). Antimicrobial Effects of Plasma-Activated Simulated Seawater (PASW) on Total Coliform and *Escherichia coli* in Live Oysters during Static Depuration. Fishes.

[63] Campbell V.M., Wang Q., Hall S.G., Salvi D. (2022). Physicochemical Properties and Antimicrobial Impacts of Plasma-activated Simulated Seawater on *Escherichia coli*. JSFA Rep..

[64] Olatunde O.O., Benjakul S., Vongkamjan K. (2019). Dielectric Barrier Discharge Cold Atmospheric Plasma: Bacterial Inactivation Mechanism. J. Food Saf..

[65] Epifanio C.E., Srna R.F. (1975). Toxicity of Ammonia, Nitrite Ion, Nitrate Ion, and Orthophosphate to Mercenaria Mercenaria and Crassostrea Virginica. Mar. Biol..

[66] Oehmigen K., Hähnel M., Brandenburg R., Wilke C.h., Weltmann K.-D., von Woedtke T.h. (2010). The Role of Acidification for Antimicrobial Activity of Atmospheric Pressure Plasma in Liquids. Plasma Process. Polym..

[67] Herianto S., Hou C., Lin C., Chen H. (2021). Nonthermal Plasma-activated Water: A Comprehensive Review of This New Tool for Enhanced Food Safety and Quality. Compr. Rev. Food Sci. Food Saf..

[68] Kaushik N.K., Bhartiya P., Kaushik N., Shin Y., Nguyen L.N., Park J.S., Kim D., Choi E.H. (2023). Nitric-Oxide Enriched Plasma-Activated Water Inactivates 229E Coronavirus and Alters Antiviral Response Genes in Human Lung Host Cells. Bioact. Mater..

[69] Wolff A., Günther T., Albert T., Johne R. (2020). Effect of Sodium Chloride, Sodium Nitrite and Sodium Nitrate on the Infectivity of Hepatitis E Virus. Food Environ. Virol..

[70] Seo K., Lee J.E., Lim M.Y., Ko G. (2012). Effect of Temperature, PH, and NaCl on the Inactivation Kinetics of Murine Norovirus. J. Food Prot..

[71] Kaushik N., Mitra S., Baek E.J., Nguyen L.N., Bhartiya P., Kim J.H., Choi E.H., Kaushik N.K. (2023). The Inactivation and Destruction of Viruses by Reactive Oxygen Species Generated through Physical and Cold Atmospheric Plasma Techniques: Current Status and Perspectives. J. Adv. Res..

[72] Boulais M., Chenevert K.J., Demey A.T., Darrow E.S., Robison M.R., Roberts J.P., Volety A. (2017). Oyster Reproduction Is Compromised by Acidification Experienced Seasonally in Coastal Regions. Sci. Rep..

[73] Russell S. (2003). The Effect of Electrolyzed Oxidative Water Applied Using Electrostatic Spraying on Pathogenic and Indicator Bacteria on the Surface of Eggs. Poult. Sci..

[74] Zver M., Dobnik D., Zaplotnik R., Mozetič M., Filipić A., Primc G. (2023). Non-Thermal Plasma Inactivation of Viruses in Water Solutions. J. Water Process. Eng..

[75] Wu Y., Liang Y., Wei K., Li W., Yao M., Zhang J., Grinshpun S.A. (2015). MS2 Virus Inactivation by Atmospheric-Pressure Cold Plasma Using Different Gas Carriers and Power Levels. Appl. Environ. Microbiol..

[76] Filipić A., Dobnik D., Tušek Žnidarič M., Žegura B., Štern A., Primc G., Mozetič M., Ravnikar M., Žel J., Gutierrez Aguirre I. (2021). Inactivation of Pepper Mild Mottle Virus in Water by Cold Atmospheric Plasma. Front. Microbiol..

[77] Hanbal S.E., Takashima K., Miyashita S., Ando S., Ito K., Elsharkawy M.M., Kaneko T., Takahashi H. (2018). Atmospheric-Pressure Plasma Irradiation Can Disrupt Tobacco Mosaic Virus Particles and RNAs to Inactivate Their Infectivity. Arch. Virol..

[78] Shaffer M., Huynh K., Costantini V., Bibby K., Vinjé J. (2022). Viable Norovirus Persistence in Water Microcosms. Environ. Sci. Technol. Lett..

[79] Rexin D., Rachmadi A.T., Hewitt J. (2024). Persistence of Infectious Human Norovirus in Estuarine Water. Food Environ. Virol..

[80] Mohamed H., Nayak G., Rendine N., Wigdahl B., Krebs F.C., Bruggeman P.J., Miller V. (2021). Non-Thermal Plasma as a Novel Strategy for Treating or Preventing Viral Infection and Associated Disease. Front. Phys..

[81] Aboubakr H.A., Gangal U., Youssef M.M., Goyal S.M., Bruggeman P.J. (2016). Inactivation of Virus in Solution by Cold Atmospheric Pressure Plasma: Identification of Chemical Inactivation Pathways. J. Phys. D Appl. Phys..

[82] Yamashiro R., Misawa T., Sakudo A. (2018). Key Role of Singlet Oxygen and Peroxynitrite in Viral RNA Damage during Virucidal Effect of Plasma Torch on Feline Calicivirus. Sci. Rep..

[83] Xu D., Cui Q., Xu Y., Wang B., Tian M., Li Q., Liu Z., Liu D., Chen H., Kong M.G. (2018). Systemic Study on the Safety of Immuno-Deficient Nude Mice Treated by Atmospheric Plasma-Activated Water. Plasma Sci. Technol..

